# Blockade of CD82 by a monoclonal antibody potentiates anti-leukemia effects of AraC in vivo

**DOI:** 10.1002/cam4.482

**Published:** 2015-07-03

**Authors:** Chie Nishioka, Takayuki Ikezoe, Akihito Yokoyama

**Affiliations:** Department of Hematology and Respiratory Medicine, Kochi Medical School, Kochi UniversityNankoku, Kochi, 783-8505, Japan

**Keywords:** AML, AraC, bone marrow microenvironment, CD82

## Abstract

We recently found that CD82 inhibits matrix metalloproteinase 9 and augments adhesion of CD34^+^/CD38^−^ acute myelogenous leukemia (AML) cells to the bone marrow (BM) microenvironment. The present study found that the use of an anti-CD82 monoclonal antibody (CD82 mAb) mobilized CD34^+^ leukemia cells from BM into the peripheral blood in a humanized AML murine model. The use of CD82 mAb in combination with cytarabine (AraC) significantly prolonged survival of immunodeficient mice-bearing human AML cells than did treatment with either AraC or CD82 mAb alone. Taken together, the combination of an anti-leukemic agent and the mobilizing agent CD82 mAb may be a promising treatment strategy to treat patients with AML.

## Introduction

In acute myelogenous leukemia (AML), the bone marrow (BM) microenvironment provides the primary site of minimal residual disease after chemotherapy 1–3. AML blast cells express many of the adhesion molecules such as CXCR4, CD117, *α*4*β*1 integrin (VLA-4), and CD44, through which AML cells interact with BM stromal cells that constitute the BM microenvironment together with vascular cells, osteoblasts, and osteoclasts. The binding of AML cells to stromal cells is one of the pathogenic characteristics of acquired drug resistance 4. Overexpressed adhesion molecules, including CXCR4 and VLA-4 on leukemia cells are associated with a higher risk of relapse 5–7. The CXCR4 antagonist AMD3100 mobilized leukemia cells into the peripheral circulation and sensitized these cells to the in vivo effects of cytotoxic chemotherapy 8. In addition, blockade of VLA-4 by a specific antibody overcame the drug resistance of leukemia cells since the drug resistance was induced by the attachment of leukemia cells to fibronectin on BM stromal cells; this process was facilitated by VLA-4 expressed on the surface of leukemia cells. The use of blocking antibody against VLA-4 in combination with cytarabine (AraC) prolonged the survival of humanized AML mice than did treatment with AraC alone in vivo 5. Another study demonstrated that granulocyte colony-stimulating factor (G-CSF) treatment of BM leukemia stem cells (LSCs), which are responsible for leukemia initiation, relapse, and resistance to chemotherapy 9, significantly decreased the number of cells in the G0 phase and increased the number in the S and G2/M phase of the cell cycle. This potentiated the elimination of chemotherapy-resistant LSCs 10. Agents that promote cell cycle entry or mobilization, such as AraC, may augment the anti-leukemic effect of chemotherapy and preferentially induce apoptosis of leukemia cells in the S phase.

CD82, a member of the tetraspanin superfamily, was originally identified as an accessory molecule in T-cell activation 11. The most well-characterized function of CD82 in non-immune cells is integrin-mediated cell adhesion to the extracellular matrix 12. CD82-mediated adhesion to fibronectin is mediated by VLA-4 in hematopoietic stem/progenitor cells 13. We found that CD82 inactivates matrix metalloproteinase 9 (MMP9) and modulates adhesion of CD34^+^/CD38^−^ AML cells to the BM microenvironment. Other researchers found that downregulation of microRNA (miR)-197 inhibits migration and invasion in hepatocellular carcinoma (HCC) cells associated with upregulation of CD82 14. These observations led us to hypothesize that blockade of CD82 by an antibody would mobilize leukemic blasts into the peripheral circulation and potentiate the cytotoxic effects of anti-leukemic agents.

## Materials and Methods

### Cells

Informed written consent was obtained from each subject in accordance with the Declaration of Helsinki. After obtaining written informed consent and Kochi University Institutional Review Board approval, leukemia cells were isolated from a patient with AML having a World Health Organization (WHO) classification system subtype of minimally differentiated AML (case 1). MOLM13, a cell line of AMLM5a with FLT3/ITD, was kindly provided by Yoshinobu Matsuo (Fujisaki Cell Center, Okayama, Japan) 15.

### CD82 antibody

The binding of human anti-CD82 monoclonal antibody (mAb) (53H5) (Santa Cruz Biotechnology, Dallas, TX) to the surface of leukemia cells was confirmed by microscopy (OLYMPUS FV1000-D) (data not shown).

### Mobilization protocol

CD82 mAb (1 *μ*g) was intravenously injected into mice-bearing human AML via the tail vein. After 0, 1, 3, and 6 h injection, mobilization was analyzed using flow cytometry after staining of peripheral blood monoclonal cells (PBMCs) with human CD34 PITC-conjugated monoclonal antibody (Biolegend, San Diego, CA, USA) and human CD45 PerCP-conjugated monoclonal antibody (DAKO, Glostrup, Denmark).

### Luc-GFP vector

The MSCV-GFP-T2A-Luciferase lenti-reporter vector was purchased from System Biosciences (Mountain View, CA). Lentiviral particles were produced using the ViraPower Packaging System (Life Technologies, Carlsbad, CA) and transduced into MOLM13 cells as previously described 16.

### Bioluminescence imaging

Trafficking of leukemia cells was assessed noninvasively by bioluminescence imaging (BLI) using an IVIS 100 CCD camera (PerkinElmer, Waltham, MA). Briefly, mice were injected intraperitoneally with d-luciferin (PerkinElmer, 150 mg/kg in PBS), and images were acquired 10 min after injection. Total photon flux (photons/sec) was quantified on images using a rectangular region of interest encompassing the entire abdomen and thorax.

### Bone marrow transplantation and engraftment assay

NOD.Cg-*Rag1*^*tm1Mom*^ *Il2rg*^*tm1Wjl*^/SzJ mice (NRG mice, Stock Number 007799) were purchased from the Jackson Laboratory (Bar Harbor, ME, USA) 17 and bred in a pathogen-free environment in accordance with guidelines of the Kochi University School of Medicine. AML (1 × 10^6^ cells) and MOLM13 cells (1 × 10^7^ cells) were intravenously injected into 6-week-old mice via the tail vein and cell engraftment was analyzed using flow cytometry after staining PBMCs with human CD82 PE-conjugated monoclonal antibody (Biolegend).

### Statistical analysis

The Student's *t*-test was used to compare differences between two groups. Statistical analysis was performed to assess the difference between 2 groups under multiple conditions by one-way analysis of variance, followed by Bonferroni multiple comparison tests. All statistical analyses were carried out using PRISM statistical analysis software (GraphPad Software, Inc, San Diego, CA). Differences were considered significant when the *P*-value was <0.05, and highly significant when the *P*-value was <0.01. Mouse survival was calculated using the Kaplan–Meier method, and survival curves were compared by a log-rank test using the PRISM statistical analysis software.

## Results

### The effect of CD82 mAb on mobilization of CD34^+^ AML cells

**Figure 1:**
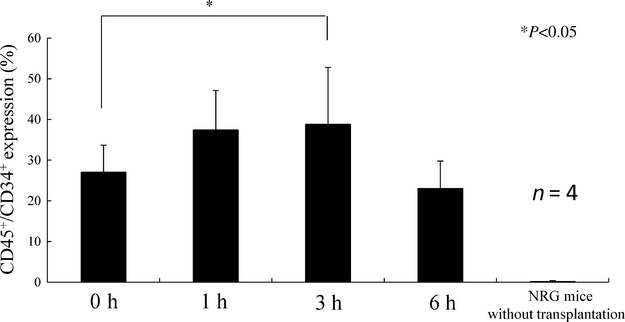
The effect of CD82 monoclonal antibody (CD82 mAb) on the mobilization of CD34^+^ cells. (A) FACS data. Gating of cell population. (B) Leukemia cells isolated from a patient with a World Health Organization (WHO) classification system subtype of minimally differentiated AML (case 1, 1 × 10^6^ cells) were intravenously injected into NOD.Cg-*Rag1*^*tm1Mom*^ *Il2rg*^*tm1Wjl*^/SzJ (NRG) mice (*n* = 4) via the tail vein. After 6 weeks, mice were treated with either CD82 mAb (1 *μ*g) or isotype control IgG. Peripheral blood samples were collected at the indicated time point (0, 1, 3, 6 h) and stained with anti-human CD45 and CD34 antibodies. NRG mice (*n* = 4) without transplantation were used as negative control. *P <* *0.05.

We examined the effect of CD82 mAb on mobilization of AML cells in vivo. We first testified if human CD82 mAb had cross-species activity with murine CD82. Western blot analyses found that human CD82 mAb reacted with lysates isolated from human AML MOLM13 cells, but not with lysates isolated from murine spleen cells that highly express CD82 (Fig. S1) 18. These observations suggested that this human CD82 antibody does not have cross-species activity with murine CD82. AML cells isolated from the patient (case 1) were transplanted into NRG mice via the tail vein. After confirming the engraftment of AML cells, CD82 mAb was intravenously injected to determine whether CD82 mAb could mobilize human CD34^+^/CD45^+^ cells into the peripheral blood (PB). Treatment of these mice with CD82 mAb increased the population of human CD34^+^/CD45^+^ cells circulating in the PB from 27% to 39% at 3 h after injection. The population of circulating CD34^+^/CD45^+^ cells subsequently decreased to baseline at 6 h after injection (Fig.1). On the other hand, the antibodies against human CD34 and CD45 antigens did not detect any CD34 and CD45 positive cells circulating in PB of NRG mice without the transplantation of human AML cells, indicating that these antibodies did not have cross-species activity with murine CD34 and CD45 antigens (Fig.1). Exposure of AML cells to CD82 mAb inhibited the survival of AML cells in vitro (data not shown), excluding the possibility that the treatment with CD82 mAb stimulated the proliferation of CD34^+^ leukemia cells in vivo. Thus, treatment of AML cells with CD82 mAb might enhance mobilization of CD34^+^ leukemia cells into the PB, although this was a transient effect. We next examined if the treatment of AML cells with CD82 mAb would potentiate the anti-leukemic effect of AraC in vivo.

### The effect of CD82 mAb and AraC on survival of AML cells in vivo

**Figure 2:**
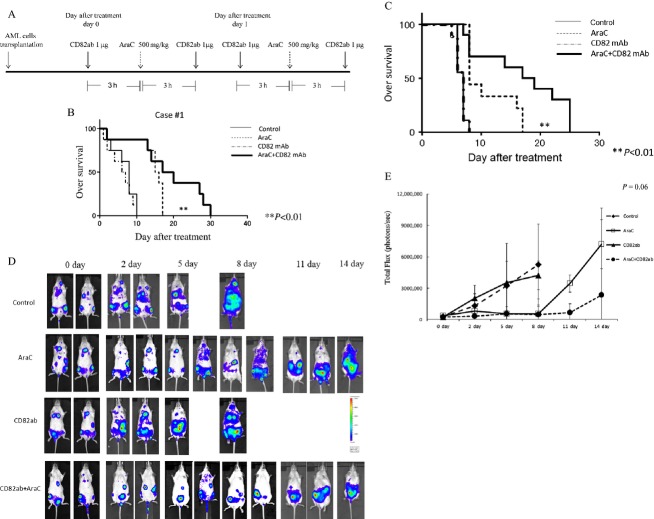
The effect of CD82 monoclonal antibody (CD82 mAb) and AraC on overall survival of human AML-bearing mice. Treatment schedule: (A) NOD.Cg-*Rag1*^*tm1Mom*^ *Il2rg*^*tm1Wjl*^/SzJ (NRG) mice-bearing human AML cells were treated with either AraC (500 mg/kg, on days 0 and 1) and/or CD82 mAb (1 *μ*g, twice a day, on days 0 and 1). The control mice received isotype control IgG. Kaplan–Meier plot of survival of leukemic mice. (B and C) NRG mice were intravenously injected with AML (case 1) cells isolated from a patient or MOLM13 cells. The NRG mice-bearing human AML cells were treated by intravenous administration of either isotype control IgG (*n* = 8), CD82 mAb alone (*n* = 8), AraC alone (*n* = 8), or the combination of CD82 mAb and AraC (*n* = 8). Statistical significance was assessed by log-rank test. **P <* *0.01. Bioluminescence imaging (BLI). (D) MOLM13 cells transduced with MSCV-GFP-T2A-Luciferase lenti-reporter vector were intravenously injected into NRG mice via the tail vein. After 3 weeks, isotype control IgG, CD82 mAb, and/or AraC (*n* = 8) were intravenously injected into MOLM13-bearing NRG mice. Leukemic cell burden was assessed by d-luciferin injection at several time points. One representative animal from each group is shown over time. Photon flux is indicated in the color scale bar. (E) Quantitative analysis of whole-body BLI. Each bar represents the mean ± SD. Statistical analysis was performed by one-way ANOVA, followed by Bonferroni multiple comparison tests.

Mice were treated 6 weeks after transplantation of AML cells with either AraC (*n* = 8, 500 mg/kg, on days 0 and 1) and/or CD82 mAb (*n* = 8, 1 *μ*g, twice a day, on days 0 and 1) (Fig.2A); control mice received isotype control IgG (*n* = 8, 1 *μ*g, twice a day, on days 0 and 1). The treatment of AML-bearing mice with AraC alone slightly prolonged their survival (Fig.2B). Notably, the treatment of AML-bearing mice with the combination of AraC and CD82 mAb significantly prolonged their survival as compared with that of mice who received AraC alone (Fig.2B). Similarly, MOLM13 AML cell-bearing NRG mice treated with the combination of AraC and CD82 mAb survived significantly longer than those treated with either AraC or CD82 mAb alone (Fig.2C). All mice that received either control IgG or anti-CD82 mAb (*n* = 8, 1 *μ*g, on days 0 and 1) died within 10 days after initiation of treatment (Fig.2C). To efficiently track leukemia cells, MOLM13 cells transduced with MSCV-GFP-T2A-Luciferase lenti-reporter vector were transplanted into NRG mice. MOLM13 AML tumor burden was traced by whole-body BLI every 3 days (Fig.2D). After 3 weeks of transplantation, measurable BLI signal was noted in all mice and treatment was initiated. The BLI signal markedly increased in both control and CD82 mAb receiving mice on day 2 and afterwards (Fig.2D). BLI signal intensity steeply increased in mice receiving AraC alone at day 11 and afterwards. In contrast, an increase in BLI intensity was less significant in mice receiving the combination of AraC and CD82 mAb (Fig.2D and E). None of the mice treated with combination of AraC and CD82 mAb showed the sign of illness and significant weight loss (data not shown).

## Discussion

This study showed that treatment with CD82 mAb increased the population of human CD34^+^ leukemia cells circulating in the PB in vivo. These observations are consistent with results of previous studies, in which treatment of human CD34^+^ cells with CD82 mAb (Abcam, Cambridge, UK) inhibited adhesion of CD34^+^ cells in the BM microenvironment 19. Moreover, CD82 mAb enhanced the anti-leukemic effect of AraC in vivo. Other investigations showed that treatment of mice transplanted with acute promyelocytic leukemia cells with AMD3100 significantly enhanced the efficacy of the anti-leukemic agent AraC 20. We found that downregulation of CD82 by shRNAs decreased the level of CXCR4 in CD34^+^/CD38^−^ AML cells (data not shown). In contrast, forced-expression of CD82 in these cells increased the level of CXCR4 (data not shown), suggesting that CD82 positively regulated the expression of CXCR4. Moreover, downregulation of CD82 increased the levels of MMP9 in AML cells 21. Thus, blockade of CD82 might augment the levels of MMP9 and CXCR4, resulting in mobilization of leukemia cells into the peripheral circulation. In addition, CD82 supports the survival of CD34^+^/CD38^−^ AML cells via the IL-10/STAT5 signaling pathway 16,21. While treatment with CD82 mAb alone did not affect the survival of AML cells in vivo (Fig.2C), the use of CD82 mAb inhibited survival of these cells in vitro (data not shown). It is possible that the dose of CD82 mAb used in this study was too low to produce anti-leukemic effects in vivo.

Taken together, these data suggest that blockade of CD82 may augment the effect of chemotherapy and could be a promising treatment strategy in individuals with AML.

## Supporting Information

**Figure S1.**CD82 expression. MOLM13 cells and spleen cells isolated from C57BL/6 mice were subjected to western blot analysis to monitor the levels of the indicated proteins. Each lane was loaded with 30 *μ*g of whole protein lysate.
