# Supplementary figures and images for: Blockade of CD82 by a monoclonal antibody potentiates anti-leukemia effects of AraC in vivo
Source: Cancer Med. 2015 Jul 3;4(9):1426–31. doi: 10.1002/cam4.482 (PMC4567027; doi:10.1002/cam4.482)

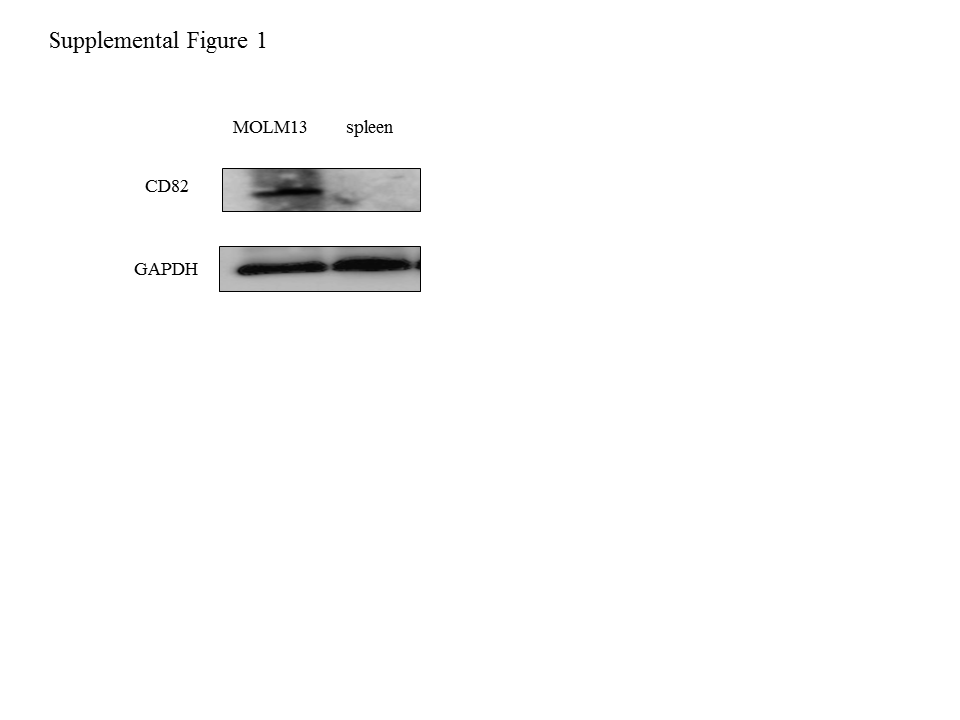

Supplement: Supplementary file 1 — Figure S1.CD82 expression. MOLM13 cells and spleen cells isolated from C57BL/6 mice were subjected to western blot analysis to monitor the levels of the indicated proteins. Each lane was loaded with 30 μg of whole protein lysate. [file cam40004-1426-sd1.tif]
